# Mutant Allele Frequency-Based Intra-Tumoral Genetic Heterogeneity Related to the Tumor Shrinkage Mode After Neoadjuvant Chemotherapy in Breast Cancer Patients

**DOI:** 10.3389/fmed.2021.651904

**Published:** 2021-03-31

**Authors:** Chun-hui Zheng, Zhao-yun Liu, Chen-xi Yuan, Xiao-yun Dong, Hai-mei Li, Jin-jin Wang, Zhao-peng Zhang, Hong-Ying Liu, Xiao-yan Ding, Wendy Wu, Rui-ru Wang, Yong-sheng Wang

**Affiliations:** ^1^Breast Cancer Center, Shandong Cancer Hospital, Cheeloo College of Medicine, Shandong University, Jinan, China; ^2^Departments of Oncology Surgery, People's Hospital, Weifang, China; ^3^Breast Cancer Center, Shandong Cancer Hospital and Institute, Shandong First Medical University, Shandong Academy of Medical Sciences, Jinan, China; ^4^Department of Radiation Oncology, The Affiliated Yantai Yuhuangding Hospital of Qingdao University, Yantai, China; ^5^Genetics Department in School of Bioscience and Technology, Weifang Medical University, Weifang, China; ^6^Department of Molecular Biology in School of Bioscience and Technology, Weifang Medical University, Weifang, China; ^7^Berry Oncology Corporation, Digital Fujian Park, Fuzhou, China; ^8^Department of Laboratory Medicine, Key Laboratory of Clinical Laboratory Diagnostics in Universities of Shandong, Weifang Medical University, Weifang, China

**Keywords:** breast cancer, next generation sequencing, gene heterogeneity, shrinkage mode, neoadjuvant chemotherapy

## Abstract

The shrinkage mode of tumor extent after neoadjuvant chemotherapy (NAC) is an important index to evaluate the odds of breast-conserving surgery. However, there is no sufficient measurement to predict the shrinkage mode after NAC. In this study, we analyzed 24 patients' formalin-fixed, paraffin-embedded samples before and after treatment and analyzed 456 cancer-related genes panel by using target next-generation sequencing. Meanwhile, the pathological shrinkage mode was reconstructed in three dimensions after surgery, and the genetic heterogeneity level was estimated by mutant-allele tumor heterogeneity (MATH). We measured the genetic intra-tumor heterogeneity and explored its correlation with the shrinkage mode after NAC. A total of 17 matched pair samples of primary tumor tissue and residual tumor tissue were successfully accessed. It was found that the most common mutated genes were TP53 and PIK3CA in both samples before and after NAC, and no recurrent mutations were significantly associated with the shrinkage mode. Besides, the MATH value of formalin-fixed, paraffin-embedded samples before and after NAC was analyzed by the area under the curve of the receiver operating characteristic, and it is feasible to classify patients into concentric shrinkage mode and non-concentric shrinkage mode in NAC based on the MATH threshold of 58. Our findings indicate that the MATH value was associated with the shrinkage mode of breast cancer in a non-linear model. Patients with the MATH value below the threshold of 58 before and after NAC displayed a concentric shrinkage mode. The area under the curve was 0.89, with a sensitivity of 0.69 and specificity of 1. Our study might provide a promising application of intra-tumor heterogeneity that is measured by MATH to make a choice of surgery.

## Introduction

Neoadjuvant chemotherapy (NAC) is prescribed increasingly in patients with advanced breast cancer (1). Previous studies have shown that NAC could facilitate breast conservation in locally advanced breast cancer, reducing volume resection in breast-conserving therapies (2, 3). In contrast, tumors downsized by NAC were reported to have a higher local recurrence after breast-conserving therapy than those who have not (4). Furthermore, large lumpectomy volumes were sacrificed in high response patients (5). There are two states of tumor shrink after NAC, concentric shrinkage mode (CSM) and non-concentric shrinkage mode (NCSM). Patients with CSM after NAC is considered to be ideal candidates for breast-conserving treatment (BCT). Simultaneously, NCSM can potentially lead to false-negative reporting of margins, which may increase the risk of locoregional recurrence (6). At the St. Gallen International Expert Consensus Conference on the Primary Therapy of Early Breast Cancer 2017, experts voted that different surgical strategies should be adopted for breast cancer based on shrinkage mode (7). It is a critical NAC therapeutic effect evaluation criterion to predict the patient will present CSM or NCSM after NAC; predictive measurements are urgently needed to inform the design of the surgical scheme and treatment strategy.

Indeed, the current method of estimating the shrinkage mode after NAC is still under research. Previous studies have reported that tumor response to NAC varied greatly by clinicopathologic variables [i.e., molecular subtype (8); clinical stage (9)]. Patients with triple-negative- and human epidermal growth factor receptor 2-positive tumors have a higher probability of achieving CSM (8). In contrast, there is still a lack of genetic composition studies to improve the stratification.

Recently, remarkable advances in oncogene investigations have made it possible to incorporate next-generation sequencing (NGS) technology into precise clinical care. Intra-tumoral genetic heterogeneity based on the NGS approach for monitoring the response to chemotherapies is currently underway. Tumors with high genetic heterogeneity were thought to contain more comprehensive resistant populations and distinct subpopulations leading to worse survival (10, 11). Thus, we speculated that the complex genetic composition might also influence the shrinkage mode after NAC. A better understanding of the genomics information of the shrinkage mode may suggest more clear classification methods for surgical strategy.

Several bioinformatics methods based on NGS have been proposed to explore tumor genetic heterogeneity (12–14). Mutant-allele tumor heterogeneity (MATH) was used to generally measure genetic heterogeneity, which is based on estimating allelic frequencies of the tumor and making it a measurable variable (15).

This study was designed to identify the pathological shrinkage modes after NAC for breast carcinomas using NGS genomic profiling. As these are clinical data regarding the response, the intra-tumor genetic heterogeneity may help to understand breast cancer biology and performing local-regional management.

## Methods

### Patients and Samples

Between October 2016 and April 2018, 24 patients with primary invasive breast cancer confirmed by histopathology with clinical stages II and III were enrolled. Breast MRI was performed before NAC and a week before surgery. Patients diagnosed with the distant metastatic disease before surgery or not examined using MRI before NAC were excluded. All the patients received a full course of anthracycline and taxane-based chemotherapy regimens and underwent radical surgery. Trastuzumab treatment was delivered to human epidermal growth factor receptor 2-positive patients.

To define the tumor molecular subtypes, we identified the immunohistochemistry (IHC) expression of estrogen receptor, progesterone receptor, HER2 status, and proliferation index (Ki-67). Fluorescence *in situ* hybridization was conducted when HER2 expression was detected as grade 2 on IHC. One percent expression rate was used as the cutoff to define positive hormone receptors. HER2 receptor was considered to be positive when HER2 expression was detected as grade 3 on IHC or HER2 gene amplification on fluorescence *in situ* hybridization. The expression of Ki-67 over 20% was considered as high.

The retrospective study was approved by the Institute Review Board of Shandong Cancer Hospital (institute review board approval number: SDTHEC201802002). The informed consents were obtained from all patients.

### Pathological Three-Dimensional Reconstruction

After a mastectomy, all specimens were cut into successive large slices and stained with hematoxylin–eosin staining. The residual tumor in the slice was outlined under the microscope, and a three-dimensional model was created under Photoshop 13.0 and 3D-DOCTOR 4.0 software (16).

### Clinical–Pathological Patterns

We divided shrinkage mode into two modes, CSM and NCSM (16).

**Figure 1:**
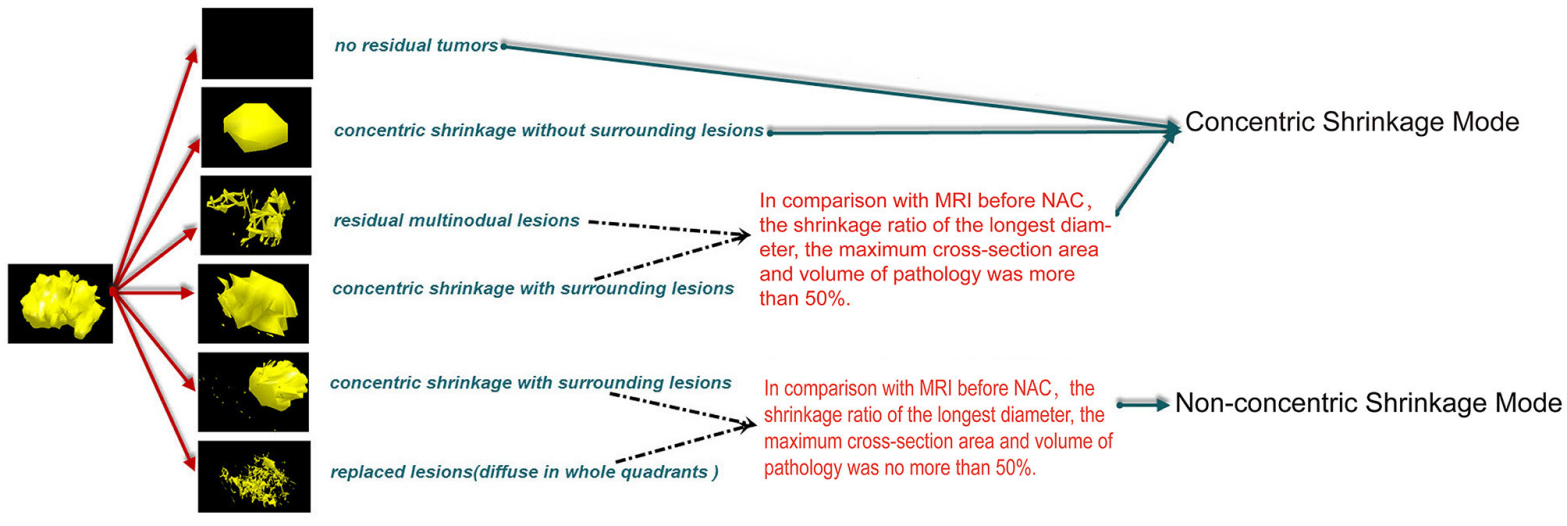
CSM means that the longest diameter retraction rates of the residual tumor were ≤ 2 cm and ≥50% compared with the longest diameter of the primary tumor before NAC and included four modes, PCR, pathologic complete response; isolated, concentric shrinkage without surrounding lesions; nodular, residual multinodular lesions; clumps with scattered, concentric shrinkage with surrounding lesions. NCSM means that the longest diameter retraction rates of residual tumor after NAC were >2 cm and/or <50% compared with the longest diameter of the primary tumor before NAC and included two modes, isolated, concentric shrinkage with surrounding lesions; clumps with scattered and diffuse replaced lesions (diffuse in whole quadrants). CSM, concentric shrinkage mode; NAC, neo-adjuvant chemotherapy; PCR, pathologic complete response; NCSM, non-concentric shrinkage mode.

MRI was performed at baseline before NAC; CSM means that the longest diameter retraction rate of the residual tumor was ≤ 2 cm and ≥50% compared with the longest diameter of the primary tumor before NAC and included four modes, pathologic complete response (PCR); isolated, concentric shrinkage without surrounding lesions; nodular, residual multinodular lesions; and clumps with scattered, concentric shrinkage with surrounding lesions. NCSM means that the longest diameter retraction rate of residual tumor after NAC was >2 cm and/or <50% compared with the longest diameter of the primary tumor before NAC and included two modes, isolated, concentric shrinkage with surrounding lesions; and clumps with scattered and diffuse, replaced lesions (diffuse in whole quadrants) (Figure 1) (16).

### Tissue DNA Isolation and Purification

Genomic DNA was extracted from the FFPE samples using GeneRead DNA FFPE Kit (Qiagen, USA). The quality of purified DNA was assayed by gel electrophoresis and quantified by Qubit® 4.0 Fluorometer (Life Technologies, USA).

### Library Construction and 456 Gene Panel Sequencing

The purified genomic DNA was first fragmented into DNA pieces around 200–300 bp using the enzymatic method (5 × WGS Fragmentation Mix, Qiagen, USA). After end repairing, a tailing and T-adaptors ligating by polymerase chain reaction (PCR) reagents were performed in pre-library. The products were followed by exon capture. Captured fragments were subsequently purified and hybridized by 456 gene panels designed by Berry Oncology Corporation, including drug-target genes and hot-spot mutated genes related to cancer development. SNV, Indel, gene fusion, and copy number variation data were detected through 456 panels in 1,000 × coverage. All results were annotated by COSMIC, TCGA, ClinVar, and in-house Berry Oncology database (Berry Oncology Corporation, Supplementary Table 1).

### Bioinformatics Analysis of Mutations

FASTP (17) was used to trim adapters and to remove low-quality sequences to obtain clean reads. The clean reads aligned to Ensemble GRCh37/hg19 reference genome performed by BWA (18). PCR duplications were processed by gencore (19). SAMtools (20) was applied to detect single-nucleotide variations (SNVs), insertions, and deletions. HGVS variant description was annotated by ANNOVAR (21) software. We excluded SNVs with PopFreqMax > 0.05 and identified non-synonymous SNVs with VAF > 0.5% or with VAF > 0.1% in cancer hotspots for further analysis.

### Statistical Analysis

Mroz et al. (15) introduced a measurement of heterogeneity termed as MATH. It is the ratio of scaled median absolute deviation (MAD) to median stated in percentage. To investigate the correlation between intra-tumoral genetic heterogeneity and the shrinkage mode after NAC, we used MATH value to assess intra-tumoral genetic heterogeneity.

The MATH value for tumors was based on the distribution of mutant-allele fractions among specific mutated loci, calculated as the percentage ratio of the width (MAD scaled by a constant factor so that the expected MAD of a sample from a normal distribution equals the standard deviation) to the center (median) of its distribution (15):

$$\begin{array}{l} MATH=100*MAD/median. \end{array}$$

The Fisher exact test was used for the comparison of MATH value between CSM and NCSM groups. *P* < 0.05 was considered statistically significant. MATH value was assessed using the area under the curve of the receiver operating characteristic (ROC). All statistical analyses were performed by SPSS 22.0 software.

## Results

We carried out high-coverage sequencing of 456 cancer-relevant genes on a group of 24 paired pre/post-NAC matched samples. Seven paired specimens (7/24, 29.1%) were excluded from the analysis because of insufficient amount/poor quality of DNA content. For 17 (17/24, 70.8%) patients, both primary tumor biopsy and residual tissues were successfully accessed and analyzed. Thirteen patients got CSM, and four patients got NCSM. Patient's characteristics are listed in Table 1.

**Table 1:** Patient and tumor characteristics.

**Pre-NAC sample No**	**Post-NAC sample No**	**Pre-MATH value**	**Post-MATH value**	**Molecular subtype**	**Shrinkage mode**
C1803725	C1803700	16.41	1.1	Luminal B/HER2 positive	CSM
C1803704	C1803772	79.74	128	Luminal B	NSCM
C1803878	C1803788	27.4	93.22	Triple-negative	CSM
C1803804	C1803818	92.47	15.3	Luminal B	CSM
C1803790	C1803821	59.7	102.7	Luminal A	NCSM
C1803787	C1803826	70.21	115.7	Her-2 positive	NCSM
C1803814	C1803829	93.11	0	Triple-negative	CSM
C1803796	C1803830	24.36	71	Luminal A	CSM
C1803799	C1803833	21.42	85.32	Luminal A	CSM
C1803870	C1803834	48.5	66.29	Luminal B/HER2 positive	CSM
C1803783	C1803840	136.3	130.1	Luminal B	NCSM
C1803813	C1803841	57.84	39.66	Luminal B/HER2 positive	CSM
C1803792	C1803854	36.1	24.68	Luminal B/HER2 positive	CSM
C1803817	C1803862	0	0	Her-2 positive	CSM
C1803805	C1803863	42.74	47.38	Her-2 positive	CSM
C1803791	C1803869	79.36	46.86	Luminal A	CSM
C1803876	C1803873	9.8	121.55	Her-2 positive	CSM

*NAC, neo-adjuvant chemotherapy; MATH, mutant-allele tumor heterogeneity; CSM, concentric shrinkage mode; NCSM, non-concentric shrinkage mode*.

### Significantly Mutated Genes in the Paired Samples

**Figure 2:**
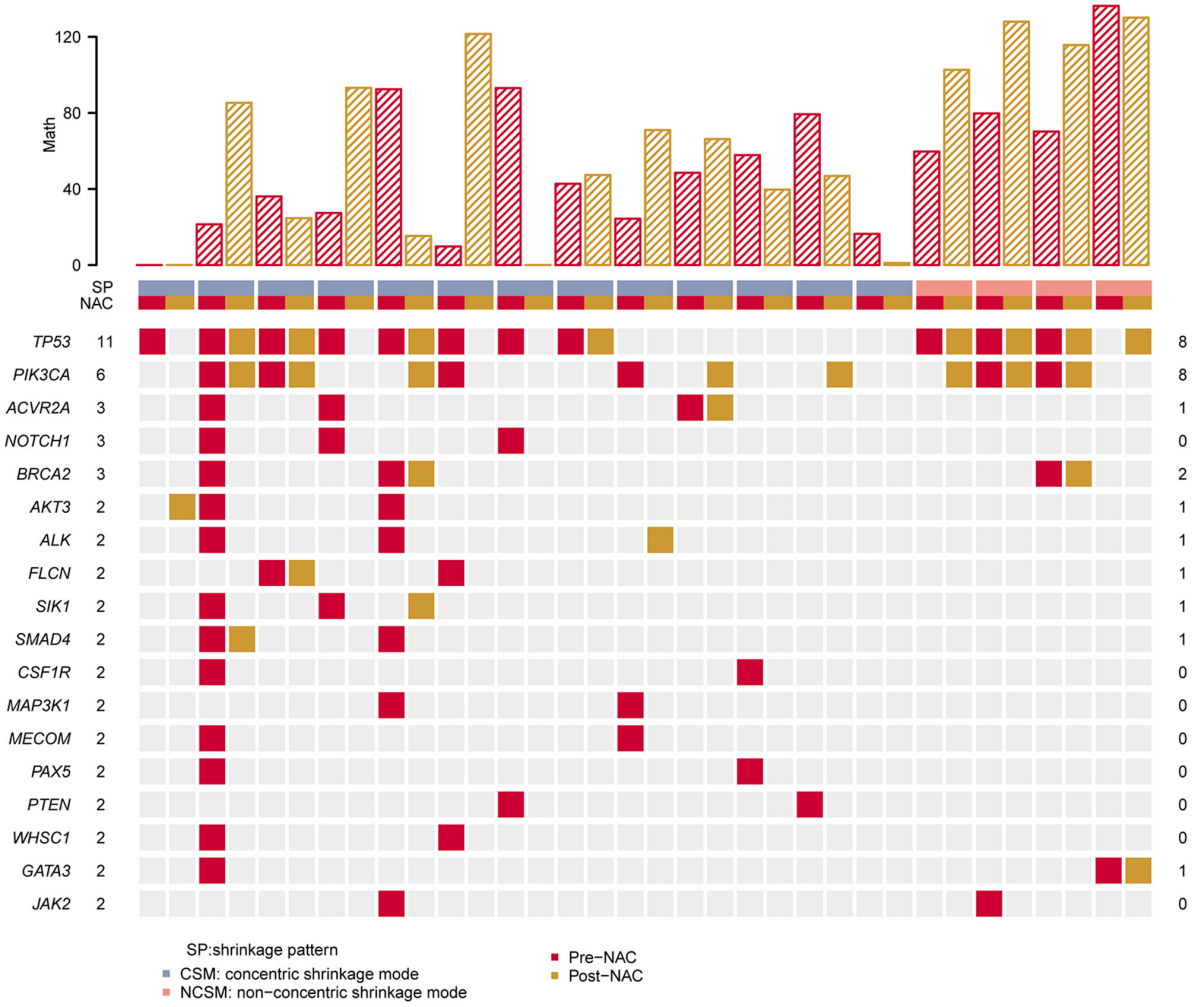
Heat map showing somatic mutation profile of CSM and NCSM. Top is the number of each MATH value of different sample. Red represents mutations detected in pre-NAC samples, and yellow shows mutations in post-NAC samples. CSM, concentric shrinkage mode; NCSM, non-concentric shrinkage mode; NAC, neo-adjuvant chemotherapy.

After NAC, the 18 most commonly mutated genes in the pre-NAC tumors were observed in this breast cancer cohort, as summarized in Figure 2. We compared the gene mutation frequency before and after NAC. In all cases, the most frequently altered genes were TP53 altered in 11 cases (11/17, 70.6%) of pre-NAC and 8 cases (8/17, 52.9%) of post-NAC samples, *PIK3CA* altered in 6 (6/17, 35.3%) of pre-NAC and 8 (8/17,47.1%) of post-NAC samples, followed by *ACVR2A* (3/17, 17.6% vs. 1/17, 5.9%), and *BRCA2* (3/17, 17.6% vs. 2/17, 11.8%) (Figure 2, Supplementary Table 2). There was no significant difference in the frequency of gene mutation between the two groups.

We compared *TP53* and *PIK3CA* hot mutations frequency between pre-NAC and post-NAC in CSM and NCSM. It was found that *TP53* hot mutations frequency in CSM declined from 70% in pre-NAC to 30% in post-NAC, and those values in NSCM raised from 50 to 100%. *PIK3CA* hot mutations frequency in CSM was from 23% in pre-NAC, then climbed to 38%, and in NSCM from 50 to 75% (Table 2).

**Table 2:** Hot mutations frequency before and after NAC.

	**TP53**			**PIK3CA**		
	**CSM (*N* = 13)**	**NCSM (*N* = 4)**	***p***	**CSM (*N* = 13)**	**NCSM (*N* = 4)**	***p***
Pre-NAC	9 (70%)	2 (50%)	0.584	3 (23%)	2 (50%)	0.538
Post-NAC	4 (30%)	4 (100%)	0.029	5 (38%)	3 (75%)	0.294

*TP53 hot mutations frequency in CSM was from 53% in Pre-NAC declined to 30% in Post-NAC, and in NSCM from 50% raised to 100%. PIK3CA hot mutations frequency in CSM was from 23% in Pre-NAC claimed to 38%, and in NSCM from 50% raised to 75%. CSM, concentric shrinkage mode; NCSM, non-concentric shrinkage mode; NAC, neo-adjuvant chemotherapy*.

**Figure 3:**
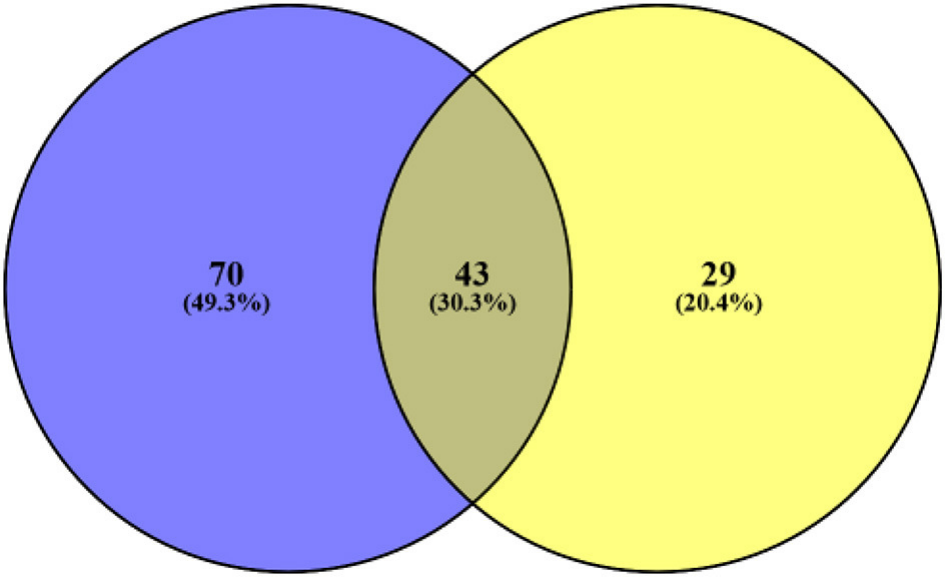
Blue represents the number of gene mutations only detected in pre-NAC, yellow represents the number of gene mutations only detected in post-NAC. Also, middle represents 43 gene mutations both in pre- and post-NAC. NAC, neo-adjuvant chemotherapy.

From all these samples, we identified 142 variants in 456 genes. One hundred thirteen mutations were detected in the pre-NAC group, and 70 gene (70/142, 49.3%) mutations were only found in the pre-NAC group (Supplementary Table 2). The mutation frequency of *NOTCH1* was 17.6% (3/17) in pre-NAC samples. Meanwhile, *CSF1R, JAK2, MAP3K1, MECOM, PAX5*, and *PTEN* were mutated in 11.8% (2/17) among pre-NAC samples, and the frequency of the remaining 63 genes was 5.9% (1/17) in pre-NAC samples. Also, these mutated genes vanished under the NAC. Concurrently, 29 gene (29/142, 20.4%) mutations were found in post-NAC samples. The frequency of these gene mutations was only 5.9% (1/17). These gene mutations were accompanied by chemotherapy, but the mutation frequency was low and scattered. There were 43 genes (43/142, 30.3%) mutated both in the pre-NAC and post-NAC samples (Figure 3, Supplementary Table 2).

### Mutant-Allele Tumor Heterogeneity Score Before and After Treatment Was Associated With the Pathological Pattern After Neoadjuvant Chemotherapy

**Figure 4:**
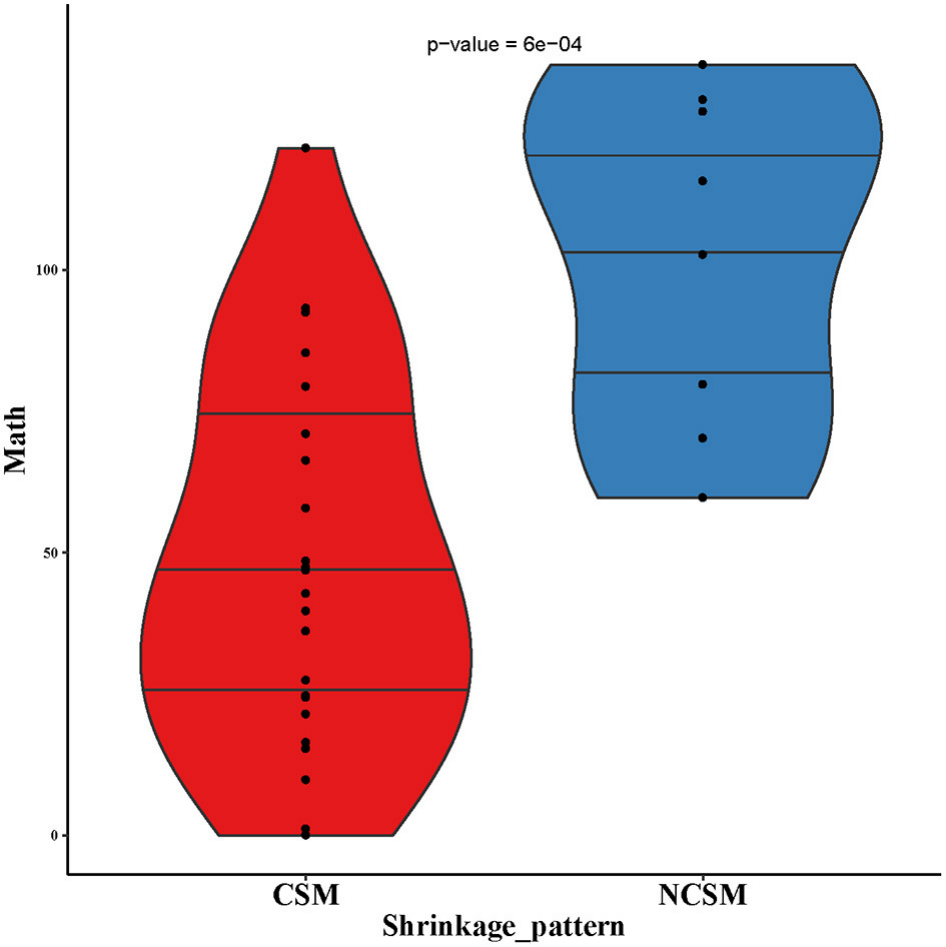
MATH values of pre-NAC in samples of CSM and NCSM. NAC, neo-adjuvant chemotherapy; CSM, concentric shrinkage mode; NCSM, non-concentric shrinkage mode.

First, we analyzed the MATH value of 17 paired samples. The MATH value of the CSM group was significantly lower than that of the NCSM group (39.66 vs. 102.7, *P* < 10–4; Figure 4). We also compared the MATH value of the samples before NAC with the samples after NAC, and the mean value of MATH in the samples before NAC was higher than that in the samples after NAC (64.05 vs. 52.67). In the CSM group, the MATH value of 6 (6/13, 46%) patients increased after NAC, whereas it was decreased in the remaining 6 (6/13, 46%) patients. What is more, we found that the mutation number before and after treatment was always one; the MATH value before and after was the same. In the NCSM group, the MATH of 3 (3/4, 75%) patients was increased, whereas it was decreased in 1 (1/4, 25%) patient (Figure 2).

**Figure 5:**
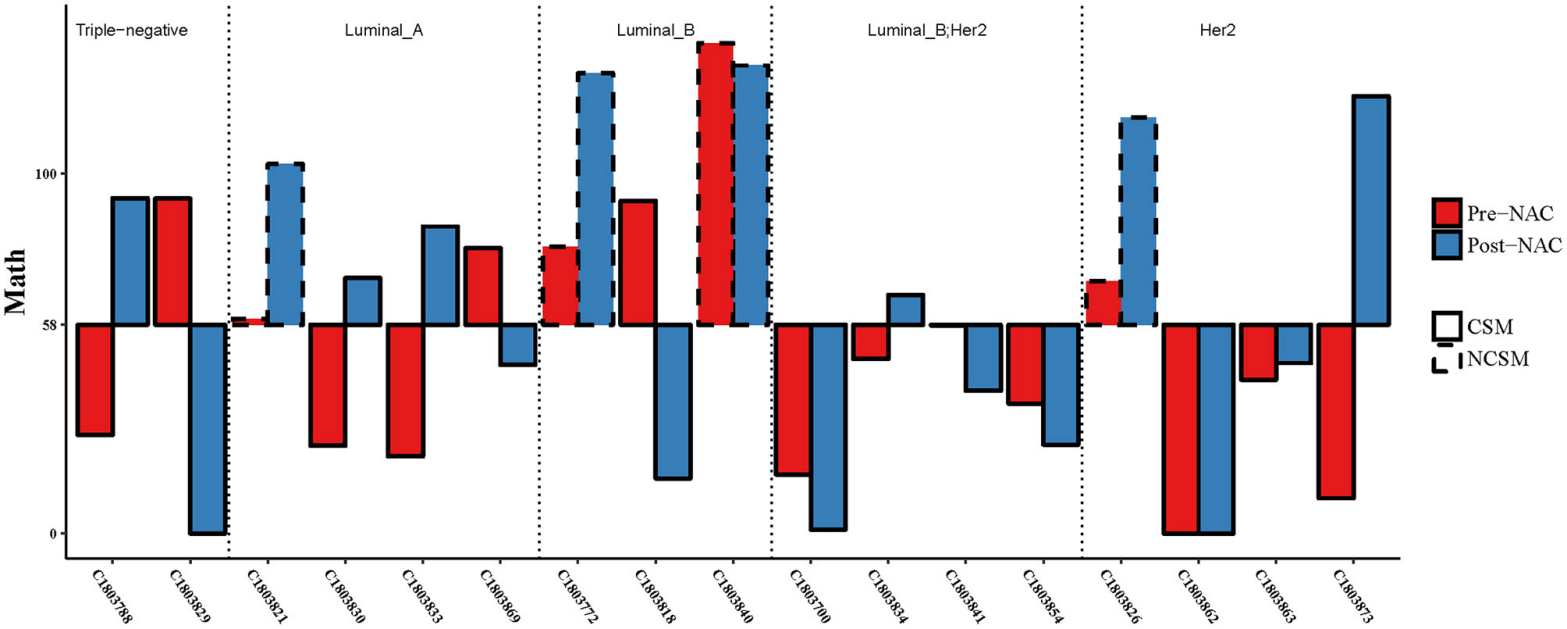
Combining the MATH values of pre- and post-NAC, when the threshold was 58, CSM and NSCM can be separated. CSM, concentric shrinkage mode; NCSM, non-concentric shrinkage mode; NAC, neo-adjuvant chemotherapy.

**Figure 6:**
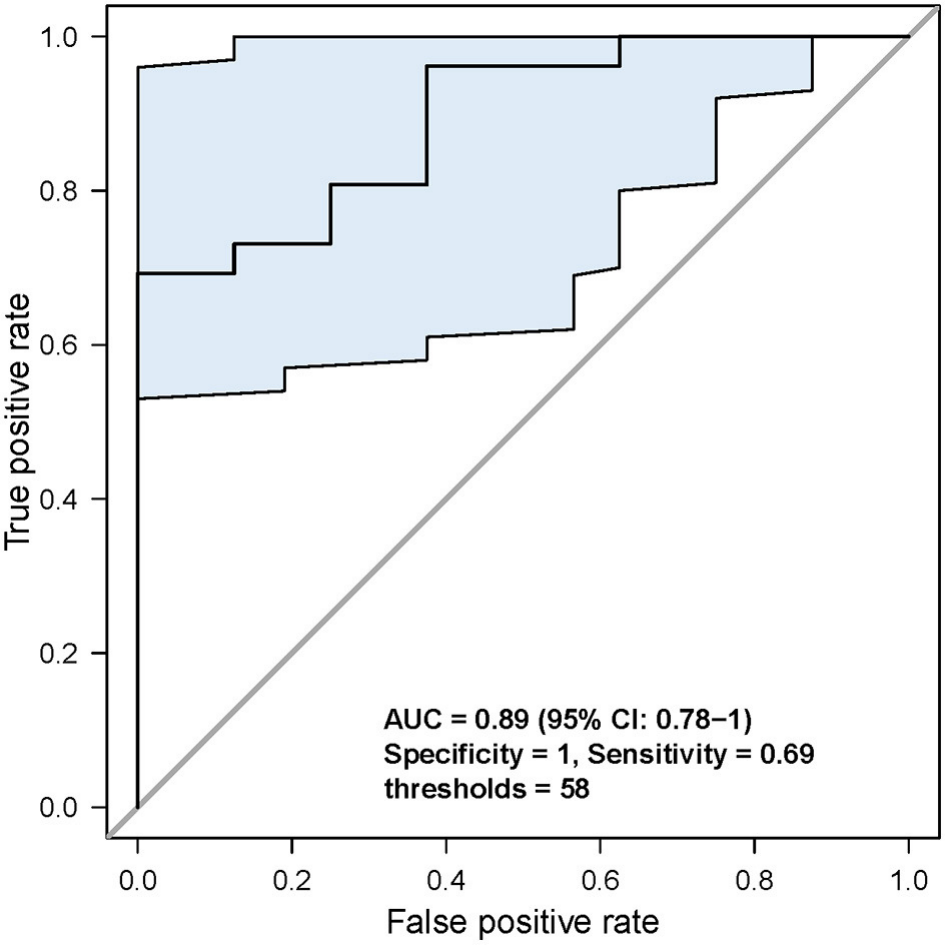
Analysis of the specificity and sensitivity of the MATH value. When threshold was 58, sensitivity and specificity were the highest.

Second, based on the analysis of the ROC curve, the optimal threshold value of MATH was 58; the tumor would have CSM after NAC, no matter the tumor samples were before NAC or after NAC (Figure 5). ROC curve was used to analyze the MATH value and shrinkage mode, as shown in Figure 6. The area under the curve was 0.89, with a sensitivity of 0.69 and specificity of 1. The threshold value of 58 indicated a good accuracy to distinguish the two different shrinkage modes.

### Characteristics of the Mutant-Allele Tumor Heterogeneity Value Before and After Neoadjuvant Chemotherapy in Patients With Different Molecular Typing

Considering the different MATH values of various molecular types of breast cancer at baseline levels, we assessed whether patients with different molecular typing met the threshold in the same way.

Three of four patients had one MATH value below the threshold of 58 (Figure 5), but the three of four patients' MATH values were increased during NAC. In the luminal B (luminal HER2-positive excluded) group, the MATH value in one of three patients was under the threshold of 58, and two of three patients declined their MATH value during NAC. In the Her2-positive group, seven of eight cases had MATH values under the threshold of 58 before NAC. In the triple-negative group, all of these patients achieved CSM, but 50% of the cases had MATH values raised after NAC. We analyzed the MATH value of the luminal B Her2-positive patients; all patients had MATH values under the threshold of 58 before NAC, and all of them achieved CSM. Obviously, if the patients had MATH values under 58, no matter what happened before or after NAC, they all would become CSM. However, not all patients with CSM would experience a reduction in MATH after NAC treatment.

## Discussion

In this study, we have detected the genomic landscape of a breast cancer cohort before and after NAC and constructed pathological three-dimensional shrinkage mode for postoperative samples. We associated the MATH value of samples before and after NAC with the shrinkage mode after NAC. Our findings extend the knowledge in the field of BCT after NAC in several ways.

First, our results may provide an effective way to select suitable patients for BCT under NAC. As we know, one issue currently under discussion during NAC is how to identify those patients who are suitable for BCT. Lack of effective measurements to select patients may lead to a decrease in BCT safety and rates. For one thing, BCT after NAC for unscreened patients may lead to an increase in local recurrence rate. A recent meta-analysis reported that NAC was associated with more frequent local recurrence than adjuvant chemotherapy: the 15-year local recurrence was 21.4% for classical treatment vs. 15.9% for neoadjuvant therapy (4). For another, considering the safety, some clinicians challenged the choice of BCT after NAC (22–24), which may be due to insufficient estimation of NCSM. Our analysis revealed that NGS of pre- and post-NAC samples might help to select patients who are suitable for BCT. However, our findings require confirmation in a larger dataset and multicenter research.

Second, our results suggested that genetic heterogeneity within a tumor may be the major factor determining the shrinkage mode after NAC. The factors affecting the shrinkage mode after NAC are currently limited to clinical parameters; genetic information was not available. Previous research by our group and others (8, 16) has investigated the association between the shrinkage mode and tumor subtype, showing that patients with triple-negative and HER2-positive tumors have a higher probability of achieving the CSM. However, we found that the shrinkage mode differed within the same subgroups; it was insufficient to match the clinical needs. In this research, our results provided direct evidence that the breast cancer patients with high-MATH before and after NAC would show NCSM.

Our results also showed that breast cancer is a highly heterogeneous disease with few high recurrent genes. In accordance with previous reports (11, 25), most genes except *TP53* and *PIK3CA* in the samples before and after NAC occurred at a low frequency. We were not able to find specific tumor somatic mutations associated with the shrinkage mode after NAC. However, our results showed that patients with CSM had a greater reduction in the number of mutations after NAC. Previous reports (26, 27) have suggested that changes in mutations may be linked to the sensitivity of chemotherapeutic drugs. Drawing on the report mentioned earlier, we guessed the possible explanation. As known, tumors with higher heterogeneity contain more variety clones than homogeneous ones. The patients with low MATH values have a relatively uniform response and sensibility to NAC and are prone to CSM. For the patients with high-MATH value, the tumor contains more variety clones; a portion of their cancer cells are sensitive to NAC, whereas the rest are not. Therefore, cells containing mutations are eliminated, and few mutations emerge, the mutant alleles relatively reduce, the patients show CSM, and the MATH values decrease below the threshold. Conversely, for patients with NCSM, their cancer cells might be resistant to NAC, and without enough loss of mutations after NAC, the mutant alleles remain or are not sufficiently reduced.

These results also raised questions, whether the heterogeneity assessment of the primary tumor is sufficient to predict treatment outcome and whether the state after chemotherapy needs to be considered. In previous studies (15, 28), the heterogeneity of the primary tumor was assessed to study the correlation with treatment outcomes without considering the heterogeneity of the tumor after chemotherapy. In contrast, they did not produce the desired results. Similarly, in our study, patients with high MATH value primary tumor still achieved the desired results and even PCR, but the MATH value after NAC was consistent. Meanwhile, we also observed changes in the heterogeneity during NAC. Both pre- and post-NAC assessments of intratumoral heterogeneity may be needed to risk stratification, a conjecture which is consistent with the previous report (27). Suffered from a shortage of sample size, the determination of threshold value was rather vague. Multiple thresholds apply to our findings. In our series of results, we selected 58 as the threshold, and all of them satisfied our findings. Larger sample size and multicenter cohort are needed to help to determine threshold values.

Noteworthy, multiple types of breast cancers differed in the assessment of MATH before and after NAC. Our results showed that 75% of luminal A patients had MATH value increased after NAC, 87.5% of Her2-positive patients were below the threshold before NAC, even all the luminal B patients were above the threshold before NAC. Combined with previous reports (29), we can conclude that molecular characteristics are related to heterogeneity. Consequently, If the conclusion was confirmed, considering the costs of serial sequencing assays that preclude its clinical implication, we pointed out that precise selection sequencing is based on molecular typing. For patients with luminal B, the NGS genetic test of post-NAC specimens was first considered. Conversely, for patients with luminal A or Her2-positive, primary tumor biopsy for NGS is the first choice.

Traditionally, the pattern of residual tumor is classified into CSM and NCSM based on morphological changes showed on MRI. Of note, we used a more precise definition of CSM, as we further characterize the shrinkage mode with concentric shrinkage. This method considered the morphology of surrounding lesions as well as the extent of the residual tumors. We defined the mode with the longest diameter retraction rate of the residual tumor ≤ 2 cm and ≥50% as CSM, which was also a good candidate for BCT and regarded as a good response to NAC. The definition seemed to be consistent with “limited multifocal regression” reported by Diane C (30). In their reports, they further subdivided multifocal regression into diffuse and limited multifocal shrinkage. Similarly, their results suggest that only the diffuse multifocal shrinkage is a risk factor that portends a worse outcome rather than a limited multifocal mode. Simultaneously, a study from the Netherlands by Briete Goorts et al. reported that patients with Pinder classification 50–90% were regarded as pathological responders (31).

Our study has some limitations. First, a small sample size may mislead the correct understanding of the result and affect the precise determination of thresholds value. Second, restricted cancer gene panel may affect the calculation of MATH results, as MATH values developed based on whole-exome sequencing (15). Furthermore, more accurate methods for determining heterogeneity, including changes in copy numbers, may enhance the study's persuasiveness.

## Conclusions

NGS provides a way to show dynamics of genetic heterogeneity before and after NAC in breast cancer with different shrinkage modes, suggesting that MATH scores may correlate with pathological shrinkage mode after NAC, informing that NGS-based approaches may have the potential to be used to estimate the shrinkage mode and to enable the selection of the most appropriate patients for BCT during NAC. Specifically, our results showed that patients with CSM had a greater reduction in the number of mutations after NAC. Thus, we provide possible explanations for genetic heterogeneity associated with shrinkage mode. Finally, optimizing the choice of pre- or post-NAC samples can be selected based on individualized molecular typing. These findings might help to optimize the choice of surgical options. To enable the selection of cost-effective sequencing options and further determine the threshold, additional large clinical studies are needed.

## Data Availability Statement

The raw sequence data reported in this paper have been deposited in the Genome Sequence Archive in BIG Data Center, Beijing Institute of Genomics (BIG), Chinese Academy of Sciences, under accession number HRA000501 that can be accessed at http://bigd.big.ac.cn/gsa-human.

## Ethics Statement

The retrospective study was approved by the Institute Review Board of Shandong Cancer Hospital (IRB Approval Number: SDTHEC201802002). The informed consents were obtained from all patients. The patients/participants provided their written informed consent to participate in this study. Written informed consent was obtained from the individual(s) for the publication of any potentially identifiable images or data included in this article.

## Author Contributions

Y-sW: conceptualization, methodology, project administration, supervision, writing-original draft, and writing-review and editing. C-hZ: conceptualization, analysis, methodology, writing–original draft, and writing–review and editing. J-jW, Z-pZ, WW, and R-rW: conceptualization, project administration, supervision, writing–original draft, and literature search. X-yDo, H-mL, X-yDi, and H-YL: analysis, writing–original draft, writing–review and editing, and literature search. Z-yL and C-xY: data curation, supervision, and investigation. All authors contributed to the article and approved the submitted version.

## Conflict of Interest

J-jW, WW, and R-rW were employed by the company Berry Oncology Corporation. The remaining authors declare that the research was conducted in the absence of any commercial or financial relationships that could be construed as a potential conflict of interest.

## Abbreviations

NAC: neoadjuvant chemotherapy
ITH: intra-tumor heterogeneity
MATH: mutant-allele tumor heterogeneity
ROC: receiver operating characteristic
CSM: concentric shrinkage mode
NCSM: non-concentric shrinkage mode
FFPE: formalin-fixed paraffin-embedded
BCT: breast-conserving treatment
NGS: next-generation sequencing.
