# Supplementary material for: Mutant Allele Frequency-Based Intra-Tumoral Genetic Heterogeneity Related to the Tumor Shrinkage Mode After Neoadjuvant Chemotherapy in Breast Cancer Patients
Source: Front Med (Lausanne). 2021 Mar 31;8:651904. doi: 10.3389/fmed.2021.651904 (PMC8044356; doi:10.3389/fmed.2021.651904)
Supplement: Supplementary file 1 [file Table_1.docx]

**Supplementary table 1: 456 gene panel list**

| ABL1 | CD38 | ERG | ICOS | MITF | PIM1 | SMARCB1 |
| --- | --- | --- | --- | --- | --- | --- |
| ACVR1B | CD48 | ERRFI1 | ICOSLG | MKNK1 | PMS1 | SMC1A |
| ACVR2A | CD69 | ESR1 | ID3 | MLH1 | PMS2 | SMC3 |
| AJUBA | CD70 | ETV1 | IDH1 | MLH3 | POLD1 | SMO |
| AKT1 | CD79A | EZH2 | IDH2 | MPL | POLE | SNCAIP |
| AKT2 | CD79B | TENT5C | IGF1R | MRE11 | POLQ | SOCS1 |
| AKT3 | CD80 | FANCA | IGF2 | MSH2 | PPARG | SOX17 |
| ALK | CD86 | FANCC | IKBKE | MSH3 | PPM1D | SOX2 |
| ALOX12B | CDC73 | FANCG | IKZF1 | MSH6 | PPP2R1A | SOX9 |
| AMER1 | CDH1 | FANCL | IL7R | MST1R | PPP2R2A | SPATA2 |
| APC | CDK12 | FANCM | INPP4B | MTAP | PRDM1 | SPEN |
| AR | CDK4 | FAS | INSR | MTOR | PRF1 | SPOP |
| ARAF | CDK6 | FBXW7 | IRF2 | MUTYH | PRKAR1A | SRC |
| ARFRP1 | CDK8 | FGF10 | IRF4 | MYC | PRKCI | STAG2 |
| ARHGAP35 | CDKN1A | FGF12 | IRS2 | MYCL | PRX | STAT3 |
| ARHGEF12 | CDKN1B | FGF14 | ITK | MYCN | PTCH1 | STK11 |
| ARID1A | CDKN2A | FGF19 | JAK1 | MYD88 | PTEN | SUFU |
| ARID2 | CDKN2B | FGF23 | JAK2 | NAV3 | PTK6 | SYK |
| ARID5B | CDKN2C | FGF3 | JAK3 | NBN | PTPN11 | TAF1 |
| ASXL1 | CEBPA | FGF4 | JUN | NCOA4 | PTPRD | TAS2R38 |
| ATM | CHEK1 | FGF6 | KDM5A | NCOR1 | PTPRK | TBL1XR1 |
| ATR | CHEK2 | FGFR1 | KDM5C | NEK11 | PTPRO | TBX3 |
| ATRX | CIC | FGFR2 | KDM6A | NF1 | PTPRT | TEK |
| AURKA | CREBBP | FGFR3 | KDR（VEGFR2） | NF2 | QKI | TET1 |
| AURKB | CRIPAK | FGFR4 | KEAP1 | NFE2L2 | RAC1 | TET2 |
| AXIN1 | CRKL | FH | KEL | NFE2L3 | RAD21 | TGFB1 |
| AXIN2 | CSF1R | FLCN | KIT | NFKBIA | RAD50 | TGFBR2 |
| AXL | CSF3R | FLT1 | KITLG | NKX2-1 | RAD51 | TIPARP |
| B2M | CTAG2 | FLT3 | KLHL6 | NOTCH1 | RAD51B | TLR4 |
| B4GALT3 | CTCF | FLT4 | KMT2A | NOTCH2 | RAD51C | TNF |
| BAGE | CTLA4 | FOXA1 | KMT2B | NOTCH3 | RAD51D | TNFAIP3 |
| BAP1 | CTNNA1 | FOXA2 | KMT2C | NOTCH4 | RAD52 | TNFRSF14 |
| BARD1 | CTNNB1 | FOXL2 | KMT2D | NPM1 | RAD54L | TNFRSF18 |
| BCL2 | CUL3 | FOXO3 | KRAS | NRAS | RAF1 | TNFRSF4 |
| BCL2L1 | CUL4A | FOXP1 | LAG3 | NSD1 | RARA | TNFSF11 |
| BCL2L2 | CXCR4 | FRK | LCK | NT5C2 | RB1 | TNFSF14 |
| BCL6 | CYP17A1 | FUBP1 | LEF1 | NT5E | RBM10 | TNFSF18 |
| BCOR | DAXX | GABRA6 | LIFR | NTRK1 | RECQL | TNFSF4 |
| BCORL1 | DDR1 | GAGE1 | LIMK1 | NTRK2 | RECQL4 | TOP1 |
| BLM | DDR2 | GALNT12 | LRRK2 | NTRK3 | REL | TP53 |
| BMPR1A | DICER1 | GATA1 | LTK | P2RY8 | RET | TSC1 |
| BRAF | DIS3 | GATA2 | LYN | PALB2 | RICTOR | TSC2 |
| BRCA1 | DNMT3A | GATA3 | MAF | PRKN | RNF43 | TSHR |
| BRCA2 | DOT1L | GATA4 | MAGEA1 | PARP1 | ROS1 | TSHZ2 |
| BRD4 | EED | GATA6 | MAGEA12 | PARP2 | RPA1 | TSHZ3 |
| BRIP1 | EGFR | GID4 | MAGEA3 | PARP3 | RPA2 | TYRO3 |
| BTG1 | EGR3 | GNA11 | MAGEA4 | PAX5 | RPA3 | U2AF1 |
| BTG2 | EIF4A2 | GNA13 | MAGEC2 | PBRM1 | RPL22 | USP9X |
| BTK | ELF3 | GNAQ | MAP2K1 | PCBP1 | RPL5 | VEGFA |
| BTLA | EOMES | GNAS | MAP2K2 | PCNA | RPTOR | VEGFB |
| EMSY | EP300 | GREM1 | MAP2K4 | PDCD1 | RUNX1 | VEZF1 |
| CALR | EPCAM | GRM3 | MAP3K1 | PDCD1LG2 | SDHA | VHL |
| CARD11 | EPHA1 | GSK3B | MAP3K13 | PDGFRA | SDHB | VTCN1 |
| CASP8 | EPHA2 | H3F3A | MAPK1 | PDGFRB | SDHC | NSD2 |
| CBFB | EPHA3 | H3F3C | MAPK11 | PDK1 | SDHD | NSD3 |
| CBL | EPHB1 | HAVCR2 | MAPK8IP1 | PHF6 | SETBP1 | WT1 |
| CCND1 | EPHB4 | HDAC1 | MCL1 | PHOX2B | SETD2 | XPO1 |
| CCND2 | EPHB6 | HDAC2 | MDM2 | PIGF | SF3B1 | XRCC2 |
| CCND3 | EPPK1 | HDAC3 | MDM4 | PIK3C2B | SGK1 | ZNF217 |
| CCNE1 | ERBB2 | HDAC6 | MECOM | PIK3C2G | SH2D1A | ZNF703 |
| CD160 | ERBB3 | HGF | MED12 | PIK3CA | SIK1 | ERCC5 |
| CD22 | ERBB4 | HIST1H1C | MEF2B | PIK3CB | SIN3A | HSD3B1 |
| CD244 | ERCC2 | HIST1H2BD | MEN1 | PIK3CD | SLAMF7 | MGMT |
| CD274 | ERCC3 | HNF1A | MERTK | PIK3CG | SMAD2 | PIK3R2 |
| CD276 | ERCC4 | HRAS | MET | PIK3R1 | SMAD4 | SMARCA4 |
| CD28 |  |  |  |  |  |  |
