# Supplementary material for: Mutant Allele Frequency-Based Intra-Tumoral Genetic Heterogeneity Related to the Tumor Shrinkage Mode After Neoadjuvant Chemotherapy in Breast Cancer Patients
Source: Front Med (Lausanne). 2021 Mar 31;8:651904. doi: 10.3389/fmed.2021.651904 (PMC8044356; doi:10.3389/fmed.2021.651904)
Supplement: Supplementary file 2 [file Table_2.doc]

| Gene | Pre-NAC patient number | Pre-NAC （n/17） | Post-NAC patient number | Post-NAC (n/17) |
| --- | --- | --- | --- | --- |
| TP53 | 12 | 0.705882 | 9 | 0.529412 |
| PIK3CA | 6 | 0.352941 | 8 | 0.470588 |
| BRCA2 | 3 | 0.176471 | 2 | 0.117647 |
| ACVR2A | 3 | 0.176471 | 1 | 0.058824 |
| NOTCH1 | 3 | 0.176471 | 0 | 0 |
| AKT3 | 3 | 0.176471 | 2 | 0.117647 |
| ALK | 2 | 0.117647 | 1 | 0.058824 |
| FLCN | 2 | 0.117647 | 1 | 0.058824 |
| GATA3 | 2 | 0.117647 | 1 | 0.058824 |
| SIK1 | 2 | 0.117647 | 1 | 0.058824 |
| SMAD4 | 2 | 0.117647 | 1 | 0.058824 |
| CSF1R | 2 | 0.117647 | 0 | 0 |
| JAK2 | 2 | 0.117647 | 0 | 0 |
| MAP3K1 | 2 | 0.117647 | 0 | 0 |
| MECOM | 2 | 0.117647 | 0 | 0 |
| PAX5 | 2 | 0.117647 | 0 | 0 |
| PTEN | 2 | 0.117647 | 0 | 0 |
| WHSC1 | 2 | 0.117647 | 0 | 0 |
| FANCM | 1 | 0.058824 | 2 | 0.117647 |
| FGFR3 | 1 | 0.058824 | 2 | 0.117647 |
| KMT2A | 1 | 0.058824 | 2 | 0.117647 |
| MAPK8IP1 | 1 | 0.058824 | 2 | 0.117647 |
| ACVR1B | 1 | 0.058824 | 1 | 0.058824 |
| APC | 1 | 0.058824 | 1 | 0.058824 |
| ARHGAP35 | 1 | 0.058824 | 1 | 0.058824 |
| ATM | 1 | 0.058824 | 1 | 0.058824 |
| BRD4 | 1 | 0.058824 | 1 | 0.058824 |
| CD276 | 1 | 0.058824 | 1 | 0.058824 |
| CHEK2 | 1 | 0.058824 | 1 | 0.058824 |
| CRKL | 1 | 0.058824 | 1 | 0.058824 |
| DOT1L | 1 | 0.058824 | 1 | 0.058824 |
| EMSY | 1 | 0.058824 | 1 | 0.058824 |
| ERBB2 | 1 | 0.058824 | 1 | 0.058824 |
| FANCG | 1 | 0.058824 | 1 | 0.058824 |
| GID4 | 1 | 0.058824 | 1 | 0.058824 |
| Gene | 1 | 0.058824 | 1 | 0.058824 |
| IRS2 | 1 | 0.058824 | 1 | 0.058824 |
| KMT2B | 1 | 0.058824 | 1 | 0.058824 |
| KMT2C | 1 | 0.058824 | 1 | 0.058824 |
| MEN1 | 1 | 0.058824 | 1 | 0.058824 |
| MSH6 | 1 | 0.058824 | 1 | 0.058824 |
| NFE2L2 | 1 | 0.058824 | 1 | 0.058824 |
| NOTCH2 | 1 | 0.058824 | 1 | 0.058824 |
| PIK3C2B | 1 | 0.058824 | 1 | 0.058824 |
| PPP2R1A | 1 | 0.058824 | 1 | 0.058824 |
| PRKCI | 1 | 0.058824 | 1 | 0.058824 |
| PTPRO | 1 | 0.058824 | 1 | 0.058824 |
| SPEN | 1 | 0.058824 | 1 | 0.058824 |
| TBX3 | 1 | 0.058824 | 1 | 0.058824 |
| TNFRSF18 | 1 | 0.058824 | 1 | 0.058824 |
| TNFRSF4 | 1 | 0.058824 | 1 | 0.058824 |
| TYRO3 | 1 | 0.058824 | 1 | 0.058824 |
| AJUBA | 1 | 0.058824 | 0 | 0 |
| ARID1A | 1 | 0.058824 | 0 | 0 |
| AXL | 1 | 0.058824 | 0 | 0 |
| BMPR1A | 1 | 0.058824 | 0 | 0 |
| BRAF | 1 | 0.058824 | 0 | 0 |
| BRIP1 | 1 | 0.058824 | 0 | 0 |
| CARD11 | 1 | 0.058824 | 0 | 0 |
| CD70 | 1 | 0.058824 | 0 | 0 |
| CIC | 1 | 0.058824 | 0 | 0 |
| CYP17A1 | 1 | 0.058824 | 0 | 0 |
| DNMT3A | 1 | 0.058824 | 0 | 0 |
| EGFR | 1 | 0.058824 | 0 | 0 |
| EP300 | 1 | 0.058824 | 0 | 0 |
| EPPK1 | 1 | 0.058824 | 0 | 0 |
| ERBB3 | 1 | 0.058824 | 0 | 0 |
| FBXW7 | 1 | 0.058824 | 0 | 0 |
| FGFR4 | 1 | 0.058824 | 0 | 0 |
| FLT1 | 1 | 0.058824 | 0 | 0 |
| FLT3 | 1 | 0.058824 | 0 | 0 |
| FOXA1 | 1 | 0.058824 | 0 | 0 |
| FOXA2 | 1 | 0.058824 | 0 | 0 |
| GALNT12 | 1 | 0.058824 | 0 | 0 |
| GNA11 | 1 | 0.058824 | 0 | 0 |
| H3F3A | 1 | 0.058824 | 0 | 0 |
| ICOSLG | 1 | 0.058824 | 0 | 0 |
| IDH1 | 1 | 0.058824 | 0 | 0 |
| IRF2 | 1 | 0.058824 | 0 | 0 |
| ITK | 1 | 0.058824 | 0 | 0 |
| JAK1 | 1 | 0.058824 | 0 | 0 |
| KDM5A | 1 | 0.058824 | 0 | 0 |
| KMT2D | 1 | 0.058824 | 0 | 0 |
| KRAS | 1 | 0.058824 | 0 | 0 |
| LRRK2 | 1 | 0.058824 | 0 | 0 |
| LYN | 1 | 0.058824 | 0 | 0 |
| MAP2K4 | 1 | 0.058824 | 0 | 0 |
| MAPK1 | 1 | 0.058824 | 0 | 0 |
| MLH3 | 1 | 0.058824 | 0 | 0 |
| MPL | 1 | 0.058824 | 0 | 0 |
| MTOR | 1 | 0.058824 | 0 | 0 |
| MYCN | 1 | 0.058824 | 0 | 0 |
| NAV3 | 1 | 0.058824 | 0 | 0 |
| NEK11 | 1 | 0.058824 | 0 | 0 |
| NF1 | 1 | 0.058824 | 0 | 0 |
| NF2 | 1 | 0.058824 | 0 | 0 |
| NKX2-1 | 1 | 0.058824 | 0 | 0 |
| NOTCH4 | 1 | 0.058824 | 0 | 0 |
| PDGFRA | 1 | 0.058824 | 0 | 0 |
| PIK3CD | 1 | 0.058824 | 0 | 0 |
| PIK3R1 | 1 | 0.058824 | 0 | 0 |
| PMS2 | 1 | 0.058824 | 0 | 0 |
| POLQ | 1 | 0.058824 | 0 | 0 |
| PPM1D | 1 | 0.058824 | 0 | 0 |
| PRKAR1A | 1 | 0.058824 | 0 | 0 |
| PTCH1 | 1 | 0.058824 | 0 | 0 |
| RAC1 | 1 | 0.058824 | 0 | 0 |
| RAD51B | 1 | 0.058824 | 0 | 0 |
| RAD54L | 1 | 0.058824 | 0 | 0 |
| TET2 | 1 | 0.058824 | 0 | 0 |
| TOP1 | 1 | 0.058824 | 0 | 0 |
| TSC2 | 1 | 0.058824 | 0 | 0 |
| VEZF1 | 1 | 0.058824 | 0 | 0 |
| WHSC1L1 | 1 | 0.058824 | 0 | 0 |
| ARHGEF12 | 0 | 0 | 1 | 0.058824 |
| ATR | 0 | 0 | 1 | 0.058824 |
| AXIN1 | 0 | 0 | 1 | 0.058824 |
| CBFB | 0 | 0 | 1 | 0.058824 |
| CD69 | 0 | 0 | 1 | 0.058824 |
| CEBPA | 0 | 0 | 1 | 0.058824 |
| CREBBP | 0 | 0 | 1 | 0.058824 |
| CTNNA1 | 0 | 0 | 1 | 0.058824 |
| CUL4A | 0 | 0 | 1 | 0.058824 |
| DICER1 | 0 | 0 | 1 | 0.058824 |
| EPHA3 | 0 | 0 | 1 | 0.058824 |
| FGF19 | 0 | 0 | 1 | 0.058824 |
| FOXP1 | 0 | 0 | 1 | 0.058824 |
| GNAS | 0 | 0 | 1 | 0.058824 |
| GRM3 | 0 | 0 | 1 | 0.058824 |
| INPP4B | 0 | 0 | 1 | 0.058824 |
| KITLG | 0 | 0 | 1 | 0.058824 |
| MEF2B | 0 | 0 | 1 | 0.058824 |
| MERTK | 0 | 0 | 1 | 0.058824 |
| MRE11A | 0 | 0 | 1 | 0.058824 |
| PBRM1 | 0 | 0 | 1 | 0.058824 |
| PIK3CB | 0 | 0 | 1 | 0.058824 |
| PTK6 | 0 | 0 | 1 | 0.058824 |
| PTPRD | 0 | 0 | 1 | 0.058824 |
| RB1 | 0 | 0 | 1 | 0.058824 |
| RPL22 | 0 | 0 | 1 | 0.058824 |
| SDHC | 0 | 0 | 1 | 0.058824 |
| SF3B1 | 0 | 0 | 1 | 0.058824 |
| XPO1 | 0 | 0 | 1 | 0.058824 |
| gene | 0 | 0 | 1 | 0.058824 |

**Supplementary table 2：Gene mutation number and frequency in Pre-NAC and Post-NAC were showed in this table.**

**NAC,** **neo-adjuvant chemotherapy**
